# Reproducibility of dynamically represented acoustic lung images from healthy individuals

**DOI:** 10.1136/thx.2007.086405

**Published:** 2007-11-16

**Authors:** T M Maher, M Gat, D Allen, A Devaraj, A U Wells, D M Geddes

**Affiliations:** 1 Department of Respiratory Medicine, The Royal Brompton Hospital, London, UK; 2 Deep Breeze Ltd, North Industrial Park, Or Akiva, Israel; 3 Department of Radiology, The Royal Brompton Hospital, London, UK

## Abstract

**Background and aim:**

Acoustic lung imaging offers a unique method for visualising the lung. This study was designed to demonstrate reproducibility of acoustic lung images recorded from healthy individuals at different time points and to assess intra- and inter-rater agreement in the assessment of dynamically represented acoustic lung images.

**Methods:**

Recordings from 29 healthy volunteers were made on three separate occasions using vibration response imaging. Reproducibility was measured using quantitative, computerised assessment of vibration energy. Dynamically represented acoustic lung images were scored by six blinded raters.

**Results:**

Quantitative measurement of acoustic recordings was highly reproducible with an intraclass correlation score of 0.86 (very good agreement). Intraclass correlations for inter-rater agreement and reproducibility were 0.61 (good agreement) and 0.86 (very good agreement), respectively. There was no significant difference found between the six raters at any time point. Raters ranged from 88% to 95% in their ability to identically evaluate the different features of the same image presented to them blinded on two separate occasions.

**Conclusion:**

Acoustic lung imaging is reproducible in healthy individuals. Graphic representation of lung images can be interpreted with a high degree of accuracy by the same and by different reviewers.

Auscultation has formed a crucial element of the clinical examination since the invention of the stethoscope by Lannec in 1816. For the pulmonologist, auscultation frequently provides important diagnostic information. The stethoscope, however, is an imperfect tool. Auscultation is a subjective process dependent on the auditory acuity and clinical experience of the user. The stethoscope attenuates high frequency sounds and does not permit quantitative evaluation of breath sounds. Furthermore, accurate documentation of auscultation findings relies on the user. With the arrival of evidence based medicine, many of the tools employed in day to day clinical practice have been subjected to rigorous scientific scrutiny.^1^ ^2^ Auscultation, however, has been much harder to validate, dependent as it is on subjective interpretation of potentially variable signs and because of a lack of robust quantitative tools.

With the advent of computer based technology, a number of investigators have sought to develop systems capable of evaluating the acoustic properties of the lungs with the aim of generating objective, reproducible, quantifiable and clinically meaningful measurements.^3^^–^8 Recent improvements in computer processing power have advanced technology beyond the point of simple “computerised stethoscopes” and have created the possibility of acoustic imaging—detailing of the structure of the lung through multipoint analysis of breath sounds. Kompis and colleagues^9^ have reported an acoustic imaging technique utilising simultaneous multi-microphone recordings to assess spatial information. By this method, they generated a static representation of the acquired data in the form of a reconstructed three-dimensional image with grey scale coding. Other investigators have used a variety of methods for displaying acoustic information including power plots in the time domain, three-dimensional spectrographs with airflow and time expanded waveforms. While these methods can be useful in determining the presence of adventitious sounds, they require a high degree of technical expertise to interpret and most provide little information about the spatial distribution of breath sounds.^10^ ^11^

Reproducibility is a prerequisite to the development of a clinically useful acoustic imaging device. Mahagnah and Gavriely^12^ have reported that the spectral pattern of inspiratory, expiratory and background sounds does not differ significantly between two recording sessions. Elphick and colleagues^13^ have demonstrated good intraobserver reliability of interpretation of computerised acoustic analysis in the detection of abnormal respiratory noises in infants. Interobserver agreement, however, was poor.

Vibration response imaging (VRI) is a commercially developed acoustic lung imaging system that displays breath sound distribution as a dynamic grey scale image designed for practical clinical application.^14^ To validate the potential utility of VRI as a clinical tool, we undertook a study in healthy volunteers. The aims of this research were to demonstrate reproducibility of recordings taken from the same individual at different time points and to assess intra- and inter-rater agreement in the interpretation of dynamic acoustic lung images.

## METHODS

### Subjects

This study was approved by The Royal Brompton, Harefield and NHLI Research Ethics Committees and written consent was obtained from all participants. Thirty-one healthy volunteers were recruited from among the staff of the Royal Brompton Hospital. Volunteers were deemed healthy on the basis of clinical history and physical examination. Individuals with a history of chronic cardiorespiratory disease or recent (within the preceding 2 months) respiratory tract infection were excluded from enrolling, as were current smokers or those with more than a 5 pack-year smoking history.

### Acoustic imaging device

The VRIxp System (Deep Breeze, Or-Akiva, Israel) is a computer based acoustic lung imaging system, developed to acquire, quantify, monitor and store breath sounds, and has been described in detail elsewhere.^14^ ^15^ Briefly, the system hardware is composed of 40 active piezoelectric contact sensors and two inactive contact sensors (lateral sensor on first row of each array) (Meditron ASA, Oslo, Norway) with a linear frequency response of ±2 db in the frequency range of 50–400 Hz; sensors are assembled on two planar arrays. Contact of the sensors with the chest wall is maintained by an open system with a constant, computer controlled low vacuum (that in bench trials (data not shown) has been demonstrated not to interfere with lung sound detection). Lung sounds, recorded during 12 s of tidal breathing, are converted to digital data by a 64 multi-channel analogue to digital conversion system (16 bit) with a sampling rate of 19.2 kHz. Inspiratory and expiratory signals are analysed separately. The digitised sounds are band pass filtered between 150 and 250 Hz to reduce interference generated by chest wall movement and heart sounds.

### Presentation of dynamic images

**Figure 1:**
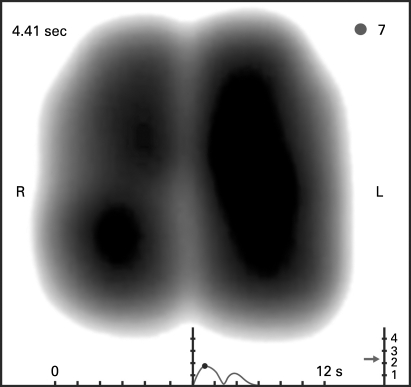
Example of a “normal” maximum energy frame—a static acoustic image equating to the period of maximal sound energy during inspiration. High intensity vibration energy is depicted in dark grey through to black. Low intensity vibration energy shows as grey and absence of vibrations as white. The graph beneath the image plots average vibration energy (first peak is maximal inspiratory energy, second peak represents maximal expiratory energy) as a function of time. The image shows smooth symmetrical lung contours with a near symmetrical distribution of acoustic lung energy. L, left; R, right.

**Figure 2:**
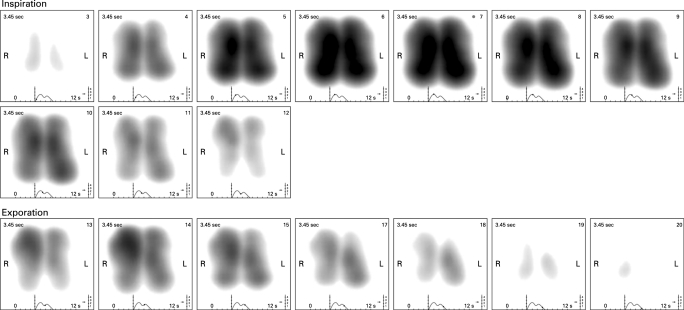
Frame by frame representation of a dynamic acoustic lung image. Each frame represents cumulative information of 0.17 s of recording time. The image demonstrates a typical crescendo–decrescendo appearance of vibration energy first in inspiration then in expiration. L, left; R, right.

Graphical representation of the filtered frequencies is by grey scale coded dynamic image, based on a two-dimensional coordinate system that corresponds with the regional position of the sensor vectors. Vibration energy is normalised across the whole recording and the output expressed on a 0–4 scale of relative intensity. High intensity vibration energy is depicted in dark grey through to black. Low intensity vibration energy shows as grey and absence of vibrations as white. In the dynamic image, the distribution of vibration energy is displayed along time. Additionally, the display shows a graph plotting average vibration energy (blue inspiration, red expiration) as a function of time. A simple user interface enables the selection and assessment of static images of vibration energy at any given point of inspiration or expiration (figs 1, 2). The image obtained at peak inspiration displays the distribution of the maximal vibration energy occurring during the respiratory cycle and has been termed the maximum energy frame (MEF) (fig 1).

### Recording procedure

Subjects were seated in a quiet environment with their hands resting on their laps. Right and left planar arrays were attached to the posterior chest wall. The arrays were positioned symmetrically with the upper row of each array approximately 2 cm above the scapula and the inner sensors of the upper rows approximately 5 cm from the vertebral column. The bottom row of each array was kept at the same height and the two arrays parallel to the vertebral column. Recordings were performed over a 12 s period while subjects took deep regular breaths at a rate of 15–20 breaths per minute. Rate and depth of breathing were guided by the investigator with reference to the dynamic images obtained. All recordings were performed by a single investigator (TMM). Subjects were recorded on three separate occasions within 1 month with at least 1 day between recordings (mean 10 (13) days). No reference was made to previous readings during subsequent recording sessions. VRI images of acceptable technical quality (as assessed by a computerised algorithm) were included from each session and used for evaluation; 87 images from 29 eligible subjects were available for analysis.

### Quantitative assessments

The sum of the signal energy for each row of both the left and right planar arrays over the full 12 s recording was used to compute an overall average for each lung field. The image was further divided into upper (rows 1–2), middle (rows 3–5) and lower (rows 6–7) zones and a relative regional assessment of the vibration energy for each of these six zones was generated. Relative regional assessment is a continuous variable (ranging between 0% and 100%).

### Qualitative assessments

Six trained raters (three pulmonologists and three radiologists) analysed representative dynamic images recorded during one complete breathing cycle taken from each of the 87 recordings available for assessment. Raters were blinded to subject identification, time point at which the image was obtained and scores given by the other raters. Raters scored each image according to: (a) the dynamic appearance, (b) frame by frame image development, (c) shape and area of maximum energy frame (the single image equating to peak inspiration), (d) overall image shape and spatial distribution of vibration energy and (e) overall impression of image. These predefined features were based on a preliminary study.^16^ These features were scored with categorical variables. In order to evaluate intra-rater reliability in the interpretation of recordings, 30 of the 87 images were randomly presented twice. Therefore, a total of 117 dynamic images, presented in the same order to each reader, were evaluated.

### Statistical methods

For qualitative assessment, the raters’ evaluations were coded and analysed by degree of consistency, agreement and reliability for each of the following categories.

1. Intra-rater reliability. For each rater and each subject, the rate of features that were evaluated identically was calculated.
2. Inter-rater agreement. For each time point, subject and feature, the evaluations that appeared most often (mode) across raters were counted and specified as the number of agreements (frequency of mode  =  f(mode)). For all of the features of each subject, the sum of f(mode) was calculated (Σf(mode)). Normalisation to 0–100% agreement level was performed (0%—no agreement; 100%—full agreement), and the average inter-rater agreement for images from 29 subjects was calculated (equation (1)).

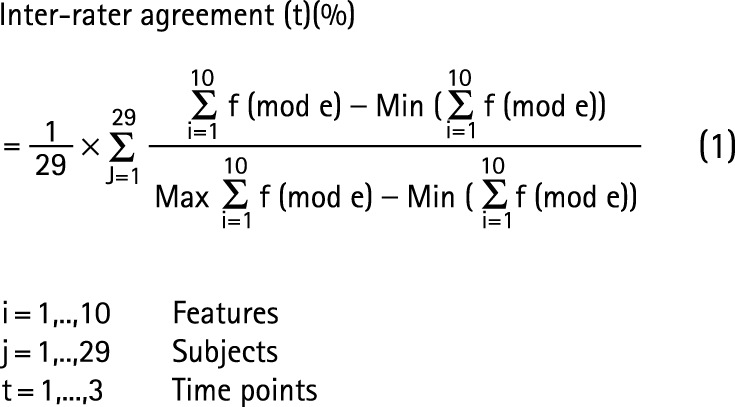


Inter-rater agreement was analysed using descriptive statistics (one way ANOVA; significance level 5%) and intraclass correlation (ICC). ICC is a quadratically modified form of the kappa correlation applied when multiple raters judge the same phenomena. A two way random effects model (considering variables of features, raters and time point) was used to calculate an averaged ICC by assuming that the ICC = ICC (subject, rater) for each time point and feature. For ICC results, positive values ranging from 0 to <0.2 indicate poor agreement, >0.2 to 0.4 fair agreement, >0.4 to 0.6 moderate agreement, >0.6 to 0.8 good agreement and >0.8 to 1 very good agreement.^17^^–^19

Reproducibility. This was calculated for each rater, subject and feature using the methodology outlined for inter-rater agreement. The minimum of Σf (mode) was 18 and the maximum 30 (equation (2)).


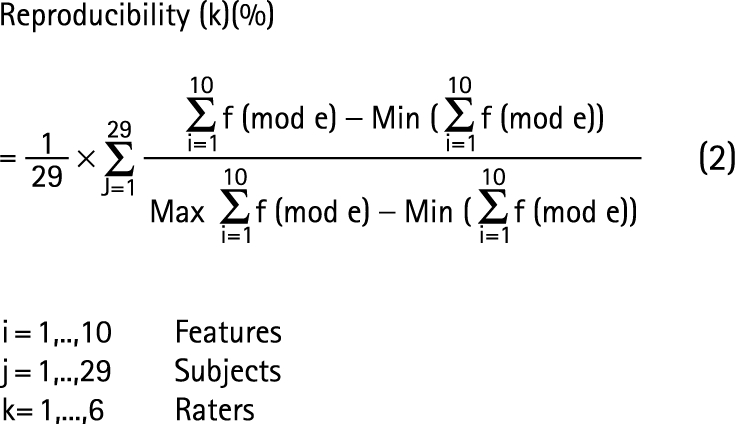


Analysis of reproducibility was performed using ICC with ICC = ICC (subject, rater, time) for each feature.

For quantitative analysis, the paired t test and one way ANOVA were used to test differences between quantitative lung values for each of the three time points. Values of p<0.05 were considered significant. The ICC model was used as ICC = ICC (subject, time) for the quantitative lung data.

## RESULTS

In all, 31 volunteers were recruited into the study. One volunteer failed to attend three recording sessions and was excluded. Recordings from one subject were excluded because of technical problems with the readings obtained. Recordings from 29 subjects (table 1) were therefore used for the purposes of this study. Males were overrepresented as a consequence of the population from which subjects were recruited. No significant differences in recordings were observed between male and female volunteers.

**Table 1:** Characteristics of the study population (n = 29)

Characteristic	
Age (y)	32 (6)
Sex (females %)	24
Height (cm)	177 (6)
Weight (kg)	78 (12)
BMI (kg/m^2^)	25 (3)
Non-smokers (%)	83
Previous smokers (%)	17

Values are mean (SD) or percentage.

BMI, body mass index.

### Normal VRI image

In the dynamic image, the image evolves centrally (presumably reflecting early sound generation in central large airways) and develops vertically in a smooth coordinated fashion. The movement of vibration energy on both the left and right is synchronous. Following peak inspiration, there is regression of vibration energy until the beginning of expiration at which point energy levels increase again until mid-expiration. Inspiratory vibration energy (as demonstrated by the line graph beneath the VRI image) is higher than expiratory energy. The MEF shape has a smooth slightly rounded contour that tapers inwards at the apex. The shape, area and image intensity of the right and left zones are near mirror images, with a tendency, however, to greater energy intensity on the left. While dynamic images from the majority of healthy individuals conformed to this pattern, there was some variability noted between images taken from different individuals (table 2).

**Table 2:** Distribution of raters’ responses for different dynamic image variables

Image feature	Variable	Response (% of total)
Dynamic image	Disturbed	26
	Good	74
Image development frame by frame	Poor	3
	Medium	3
	Good	94
MEF shape	Poor	5
	Medium	8
	Good	87
MEF area	R>L	6
	R<L	21
	R = L	73
MEF intensity	R>L	5
	R<L	31
	R = L	64
MEF missing parts right upper	No	98
	Yes	2
MEF missing parts right lower	No	78
	Yes	22
MEF missing parts left upper	No	94
	Yes	6
MEF missing parts left lower	No	95
	Yes	5
VRI final assessment	Regular	66
	Irregular	34

L, left; MEF, maximum energy frame; R, right; VRI, vibration response imaging.

### Quantitative analysis of reproducibility

The proportion of vibration energy detected over the left lung during the whole of the respiratory cycle was mean 55 (SD 5)% and the right lung 45 (5)% (table 3). The mean coefficient of variance was 5% and the mean absolute relative error 4% (95% confidence interval 2.8% to 4.5%). In 86% of the cases, the left lung was dominant. The difference between any two recordings from the same individual was less than 5% of total left or right lung energy in 76% of cases and less than 10% in 98% of cases. No significant difference (p value >0.05, one way ANOVA) was found between the average energy values for the left or right lung between the three recording time points.

**Table 3:** Distribution of regional lung energy (n = 29) at each recording

	Recording 1	Recording 2	Recording 3	Mean
Upper left	9.7 (2.9)	9.4 (1.9)	9.5 (2.5)	9.6 (2.1)
Middle left	27.3 (3.7)	26.4 (3.8)	26.8 (4.9)	26.9 (3.5)
Lower left	18.7 (5.1)	20.0 (5.1)	20.0 (5.1)	19.1 (4.7)
Upper right	7.3 (2.5)	7.1 (2.7)	7.1 (2.7)	7.2 (2.3)
Middle right	21.4 (3.9)	20.1 (4.5)	20.1 (4.5)	21.0 (3.7)
Lower right	15.3 (4.5)	17.2 (4.1)	17.2 (4.1)	16.3 (3.6)
Total left lung	56.0 (4.8)	55.8 (6.0)	54.9 (4.9)	55.4 (4.9)

Values are mean (SD).

**Figure 3:**
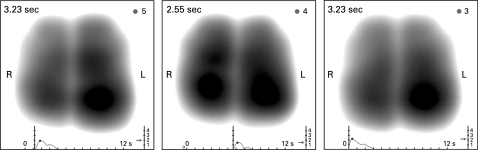
Example of the maximum energy frame taken from three separate recordings obtained from one subject. Images demonstrate similarity in shape, relative lung size and vibration energy distribution across time.

A similar degree of reproducibility was found when the lungs were assessed regionally (table 3). It is noteworthy that within a recording there was a heterogeneous distribution of the averaged vibration energy in the six different zones of the lung images. This heterogeneity was found to be repeatable during subsequent recordings (fig 3). For the six lung zones, the coefficient of variance was 13% and average absolute relative error 10 (7)%. No significant differences were found between the average relative regional assessment values for the six zones of the lung between any of the three time points (p value>0.05, one way ANOVA and t test for paired data).

ICC using a model of subject–time demonstrated very good reproducibility for both total lung (ICC 0.86) and six zone (ICC 0.83) assessment.

### Qualitative data interpretation

#### Intra-rater reliability

**Figure 4:**
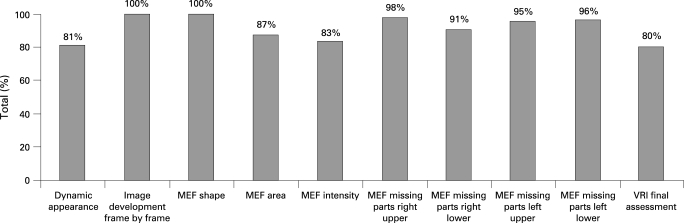
Intra-rater agreement in the interpretation of the different key features of the acoustic lung image (data based on 30 images reported twice). MEF, maximum energy frame; VRI, vibration response imaging.

The average value for overall identical evaluations of the 10 features of the VRI image scored by the raters ranged from 88% to 95% per rater. Furthermore, the percentage of identical evaluations for each individual feature by raters demonstrated a high rate of consistency (fig 4). Features interpreted with absolute agreement between raters were frame-by-frame development and MEF shape. Overall assessment of the image elicited the least agreement (80%).

#### Inter-rater agreement

**Figure 5:**
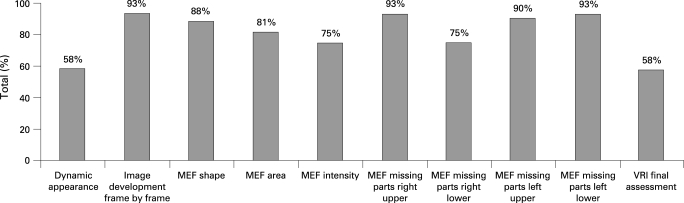
Inter-rater agreement in interpretation of acoustic lung images. Data based on the evaluation of images from all subjects at each of the three time points—a total of 87 images. MEF, maximum energy frame; VRI, vibration response imaging.

The averaged agreement, based on the 87 images from 29 subjects and three time points, was 82 (9)%. There was no significant difference found between the six raters at any of the time points (one way ANOVA, t test for paired data, p>0.05). The level of agreement varied according to the image feature evaluated (fig 5).Using a model of subject–rater for the averaged features, the ICC was 0.61 (good level of agreement).

#### Reproducibility

The degree to which raters were consistent along the three time points was examined for each rater separately. Among the six raters, the lowest value for reproducibility was 80% and the highest value was 91%. The averaged value for reproducibility of the six raters was 84 (12)%, demonstrating a very good level of consistency. There were no significant differences found between the reproducibility rate of the six raters (one way ANOVA, p>0.05). ICC using a model of subject–rater–time for the averaged features was 0.76, again demonstrating good agreement.

## DISCUSSION

Acoustic lung imaging was first envisaged over three decades ago. However, considerable hurdles have needed to be overcome to enable the concept to become reality. Lung sounds have to be accurately recorded and extraneous sounds removed. The resulting sound information needs to be presented in a clinically meaningful manner. Furthermore, results need to be accurate and reproducible. The majority of acoustic research has focused on the detection and interpretation of adventitious sounds. A small number of studies have assessed temporal consistency in breath sound recording in healthy adults.^12^ ^20^ ^21^ In the largest of these studies, Sanchez and Vizcaya^20^ recorded frequency spectra of lung sounds in 10 normal adults on seven occasions over a year using contact sensors over the suprasternal notch and the right posterior lower chest wall. No significant spectral differences between the measurements obtained from each individual were found.

The current study was designed to demonstrate that the algorithms employed by the VRIxp device and the method of displaying acoustic data as a dynamic image are capable of generating reproducible images. We showed that acoustic lung images obtained using the VRIxp are highly reproducible across recordings from the same individual made at different times when measured both quantitatively and qualitatively. Quantitatively, the degree of reproducibility, as determined by ICC, was very good for both total lung and regional lung assessment (ICC 0.86 and 0.83, respectively). We demonstrated a similar degree of reproducibility when dynamic lung images were scored qualitatively (ICC 0.76). These results are comparable to previous smaller studies.^12^ ^20^ ^21^ The subjects recruited into this study were predominantly male with a relatively low median age and normal median body mass index. We do not believe that this influenced our results. Although age and gender have previously been demonstrated to affect the spectral characteristics of lung sounds,^22^ they have not been shown to influence reproducibility.^12^ ^20^ Neither we, nor other investigators, have noted an effect of body mass index on the reproducibility of lung sounds.^20^ ^21^ We hope to explore the impact of age, gender and smoking history on acoustic lung images obtained using the VRIxp device in future research.

Analysis of the quantitative data demonstrated a heterogeneous distribution of breath sounds with a pattern of left sided dominance of vibration energy (regional assessment of vibration energy recorded throughout the respiratory cycle identified the left lung as contributing >50% of total sound energy in 86% of dynamic images (mean 55 (SD 5)%). Such asymmetry has been noted previously and has been postulated to relate to the effect of mediastinal structures and airway geometry on airflow turbulence.^22^^–^24 However, when the MEF was considered alone, the left lung was dominant in only 31% of cases. This suggests that regional differences in airflow are much less prominent at peak inspiration, possibly reflecting the greater contribution of small airways to total lung vibration energy at this stage in the breathing cycle. The heterogeneous distribution of the averaged vibration energy across the dynamic lung images was found to be repeatable during subsequent recordings. Furthermore, the relative distribution varied across each lung being greatest in the mid zones and least in the upper zones. This variation is in keeping with that observed previously by Pasterkamp *et al.*^25^ ^26^

We have also shown that the dynamic representation of acoustic lung images can be reliably interpreted both by the same individual (intra-rater reliability 88–95%) and by different individuals (inter-rater agreement 82 (9)%, ICC = 0.61). The raters used for this study were either radiologists or pulmonologists. Raters’ previous exposure to VRI images varied from little prior experience to over 1 year’s experience in interpreting both normal and pathological images. All raters received a standardised 4 h period of training on the day prior to their involvement in the study. Despite a wide range of expertise in interpreting VRI images, there was considerable agreement between raters. It is conceivable that with further experience in reporting of VRI images, the level of agreement would increase further.

The features of the acoustic lung image that were chosen for assessment were selected on the basis of earlier preliminary studies. Even among our group of young healthy adults, we observed a range of appearances in the dynamic image (table 2). The significance of this variation requires further research in a larger, more diverse cohort of healthy individuals to enable a clear characterisation of the range of “normality”. Quantitative analysis of the acoustic lung images produced results in agreement with those produced by qualitative image assessment. We observed that some features of the dynamic lung image were scored by raters with a greater degree of consistency. It is notable that the level of both intra- and interobserver agreement was greatest for assessment of static features of the acoustic images. Agreement for interpretation of the dynamic image (dynamic appearance and overall image interpretation) was less good. This may reflect raters’ greater clinical expertise in the interpretation of static images (eg, *x* rays). As raters gain experience in the reading and interpretation of dynamic lung images it is our expectation that their degree of consistency will improve further.

For this study, subjects were asked to take regular deep breaths at a respiratory rate of 12–15 breaths per minute. Subjects received feedback on their breathing intensity in the form of a graphic display and, if necessary, were asked to modify their breathing. The fact that intra-subject values were reproducible verifies the feasibility of this technique. Moreover, Gavriely and colleagues^4^ have demonstrated that increasing flow rate causes parallel upward shifts in lung sound spectral curves with no change in shape and pattern of the chest wall spectra. This observation, combined with the results of the current study, suggest that the relative distribution of normal breath sounds is not significantly affected by flow rate. This is of importance as relative breath sound distribution that varies with flow rate may be indicative of respiratory disease.^14^ We plan to explore the effects of variation in breathing depth, pattern and rate on the reproducibility of acoustic lung images in health and disease states in future studies.

We chose to validate the VRIxp in healthy individuals because of the expectation that lung sounds will change little over time. It is a necessary prerequisite for any investigative tool that it should generate reproducible data from individuals in whom there is no change in lung health status. Further work is required to investigate the differences seen with acoustic lung imaging in respiratory disease and to demonstrate that reproducible images can be obtained from individuals with stable chronic respiratory disease. A recent study by Dellinger and colleagues,^15^ using the VRIxp acoustic lung imaging system, has shown that in an intensive care setting, changes in ventilator modes have a discernible and reproducible effect on the pattern and distribution of dynamic acoustic lung images. These findings taken together with our results suggest that the VRIxp device has the ability to detect meaningful change in lung sounds. Studies have shown that normal lung sounds have distinctive characteristics that can be differentiated from abnormal lung sounds,^9^ ^26^ thus supporting the potential clinical value of acoustic lung imaging. In the future, computerised lung sound analysis may be used for the detection of the early stages of airways disease,^14^ for monitoring mechanically ventilated patients^14^ ^15^ and in measuring regional ventilation and airflow obstruction.^15^

In summary, we have demonstrated the reproducibility of acoustic lung images taken from the same individual at different times over a period of 1 month. Furthermore, we have shown that the graphical representation of dynamic acoustic lung images can be interpreted reliably by both the same rater and by different raters. We believe that with further research, acoustic lung imaging can be added to the physician’s repertoire of non-invasive tools for screening, diagnosing, monitoring and studying diseases of the lung.
